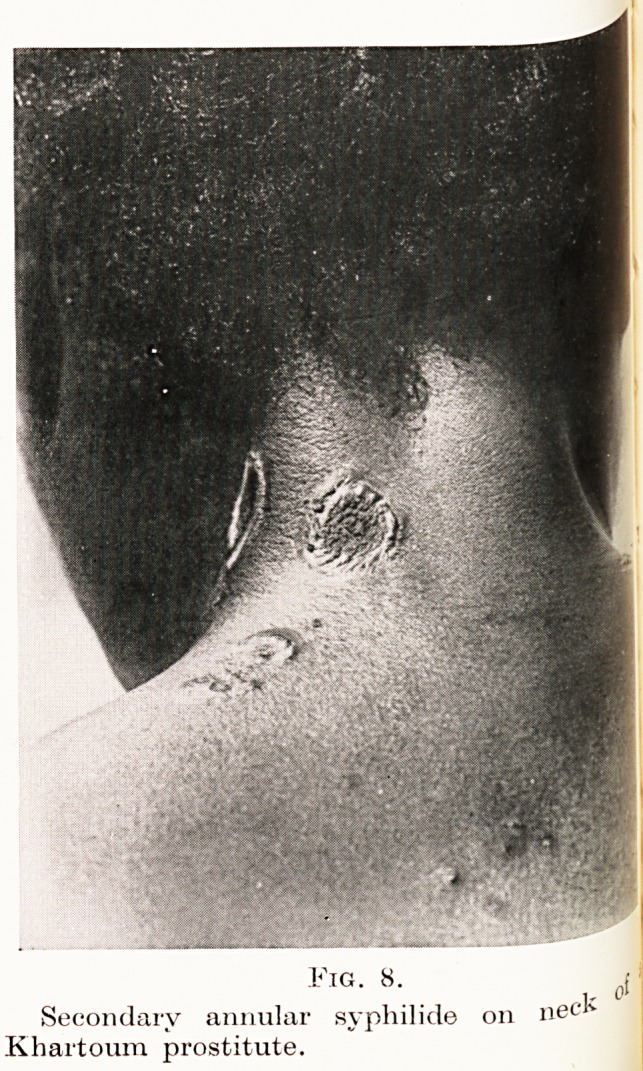# Syphilis in the Tropics
*A Paper read to the Society on Wednesday, 9th November, 1938.


**Published:** 1938

**Authors:** T. F. Hewer

**Affiliations:** Professor of Pathology, University of Bristol; Hon. Pathologist to Bristol General Hospital and Bristol Children's Hospital


### SYPHILIS IN THE TROPICS.*

BY

T. F. Hewer, M.D., M.R.C.P.,

*Professor of Pathology, University of Bristol; Hon. Pathologist to Bristol General Hospital and Bristol Children's Hospital.*

---

In the middle of the fifteenth century, when syphilis first appeared in Europe, it was known as the *great pox*, because its cutaneous manifestations so far exceeded in their severity those caused by smallpox. Since that time the disease in Europeans has gradually suffered a change in that the cutaneous lesions have become progressively less striking while the visceral damage has become more serious. To-day one seldom sees a profuse secondary syphilide in Europe, whilst subcutaneous gummata, necrosis of the bones of the skull and similar gross lesions of the tertiary stage are great rarities: but aortitis and general paresis remain to remind us of the seriousness of the disease.

It happens that in different parts of the world to-day, in different races of mankind, syphilis manifests itself in a guise reminiscent of that which it adopted in the fifteenth century in Europe. If we travel from Central Africa down the White Nile to Egypt, and then make a detour through Northern Africa as far as Morocco, returning to Palestine, Syria, and Turkey,

* A Paper read to the Society on Wednesday, 9th November, 1938.

and, finally, pass up the Dalmatian coast on our way home to Western Europe, we shall see all sorts of gradations between the syphilis of the middle ages and the disease as we know it in England to-day.

### YAWS.

Before we embark upon this journey it is necessary to decide whether we shall include in our study the tropical disease which goes by the name of *yaws*. There has long been a controversy on this subject, some maintaining that yaws is a separate entity while others consider it merely syphilis in a different soil. It may repay us to consider how the distinction between the two conditions came to be made and upon what criteria that distinction is now maintained. In the early days of the exploration of Central Africa medical observers were impressed by the prevalence of a disease which they, from their experience in Europe, recognized as syphilis; they noticed that it produced very gross cutaneous lesions of a granulomatous nature which, to their epicurean eyes, resembled raspberries: for this reason the French named it *frambœsia.* In 1902 Jonathan Hutchinson published a monograph on yaws and in this he expressed the opinion that it was syphilis modified by race and climate. Then in 1905 Schaudinn and Hoffmann described the spirochæte of syphilis, *Treponema pallidum*, which they observed in chancres and inguinal glands of syphilitic patients. Later in the same year Castellani applied Schaudinn's method to two cases of yaws in Ceylon and discovered a treponema which he considered different morphologically from that of syphilis: this was named *Treponema pertenue.* It is now known that the two organisms are indistinguishable, but this is, of course, no proof of their actual identity. Unfortunately, the mistaken belief that the treponema of yaws was proven to be a distinct organism from that of syphilis led to an almost universal acceptance of the view that the two diseases were distinct.

Certain criteria have been enunciated whereby a differential diagnosis may be made on clinical grounds. They are well summarized in the following table taken from Manson-Bahr's[1] text-book of tropical diseases :—

| YAWS. | SYPHILIS. |
|---|---|
| Not congenital. | Congenital. |
| Primary sore—extragenital. | Primary sore—usually genital. |
| *Secondary Stage.* | *Secondary Stage.* |
| (*a*) Typical yaw pathognomonic : furfuraceous desquamation and plantar lesions characteristic. | (*a*) Seldom imitates frambœsia. |
| (*b*) Mucous membranes not affected. | (*b*) Mucous membranes affected. |
| (*c*) Itching common. | (*c*) Itching rare. |
| (*d*) Alopecia unknown. | (*d*) Alopecia may occur. |
| (*e*) Eyes unaffected. | (*e*) Iritis common ; choroiditis and retinitis rare. |
| *Tertiary Stage.* | *Tertiary Stage.* |
| (*a*) Visceral lesions absent. | (*a*) Visceral lesions occur, *i.e.* peri-cellular cirrhosis, gumma of liver, kidney, etc. |
| (*b*) Nervous system never seriously affected. | (*b*) Nervous system prone to infection : Tabes, G. P. I. |
| (*c*) Blood-vessels : no endothelial proliferation as in syphilis. | (*c*) Endarteritis obliterans—cerebral thrombosis. |
| Yaws better resisted. Constitutional disturbances slight : great exuberance of eruption and cheloid scarring. | Syphilis attacks constitution, affecting the vital structures. |
| Does not respond to mercury. | Responds well to mercury. |

This table has provoked a great deal of criticism, notably that of Blacklock[2] (1932), whose analysis of the question was a great consolation to me at a time when I was beginning to despair of finding a case of true yaws.

Bearing in mind the distinctions tabulated above we will now survey briefly the manifestations of

*treponematosis*—if one may use that term to cover yaws and syphilis—in different parts of the world.

### SOUTHERN SUDAN.

The southern boundary of the Bahr-el-Ghazal province of the Anglo-Egyptian Sudan is adjacent to the Belgian and French Congos. Part of the country is mountainous with many streams, but the greater portion consists of low-lying land which is under water for many months of the year. The climate is hot and humid. The various indigenous tribes wear no clothing and live in primitive huts where close bodily contact is inevitable; they have no notion of methods of isolation of the sick, although they are usually aware that they contract diseases from their neighbours. Should one of them develop *treponematosis* and become covered with a secondary rash his relatives will still sleep and feed with him, using the same food vessels and sleeping in the same bed: it is little wonder that the disease is contracted non-venereally with extra-genital primary inoculation.

In the Bahr-el-Ghazal the disease is called yaws. Here it most nearly satisfies the criteria given above. Children are more frequently affected than adults. The primary lesion, if it can be distinguished, is usually extra-genital, and the secondary stage is characterized by a profuse cutaneous eruption of crusted papules which contain spirochætes demonstrable by dark-ground examination. Figure 1 is a photograph of the shoulder of a small boy whose cutaneous eruption had been present for two or three months. There are many large, flat, granulomatous papules, teeming with spirochætes, which in places show healing at the centre with epithelialization: such central healing gives rise eventually to annular lesions similar to that in Figure 2. It is a notable feature of these skin lesions of the secondary stage that, while the majority heal spontaneously in a few months, a few are apt to persist for a long while. It is not uncommon to see condylomata in the axillæ and perineum a year or more after all other lesions have resolved. Periostitis is a frequent occurrence in the secondary stage and is one of the most distressing features of the disease: in the absence of treatment it is apt to persist for years. For these reasons it is often impossible to decide whether the patient is in the secondary or in the tertiary stage: the latent period seen so constantly in Europe is absent.

In the absence of any facilities for autopsies one cannot give an accurate account of the visceral lesions. If aneurysms occur they are very rare. I have seen one case of aortic regurgitation in a man with a positive Kahn reaction who gave a history of having the disease several years previously; he also had leukoplakia of the tongue and some small papillomata on the scrotum. His four children, one born before he was infected, also had cutaneous lesions and were said to have caught it from the same source as their father; one of them is illustrated in Figure 2.

Not infrequently in the late stages granulomata appear beneath the skin of the sole of the foot. On account of the tension of the dense subcutaneous tissue and the thick skin in this situation these granulomata are very painful and make walking difficult. The name *crab yaws* is applied to this condition because the patient walks awkwardly, like a crab. It is indisputable that one does not see this manifestation of *treponematosis* in the northern Sudan, or in any other parts of the world where the disease is called syphilis. To my mind this phenomenon may be explained on environmental grounds: it occurs in districts where the natives walk bare-foot and where the country is swampy or actually under water for a great deal of the year. We have seen that cutaneous lesions of the secondary stage persist in moist regions of the body, and it is likely that a combination of trauma and moisture applied to the soles of the feet may explain the development of the lesions of crab yaws.

The condition known as *gangosa* (Figure 3) is a destruction of the palate and nasal bones which may occur a year or more after the onset of yaws; it is really an exaggeration of the palatal perforation seen in tertiary syphilis.

Meningo-vascular disease, tabes and general paresis are unknown: but routine examination of the cerebro-spinal fluid gives evidence of meningeal irritation—a significant increase of cells or protein—in some cases. In my series the changes were slight and far less frequent than those encountered in the northern Sudan.

Congenital transmission is rare. I saw one case of a new-born infant covered with a profuse cutaneous eruption: its mother was in the florid secondary stage. No instance of late congenital disease has ever been noted.

In summary we may say that in the southern Sudan *treponematosis* is largely a childhood disease caught by contact. The cutaneous lesions are profuse and persistent and, with the exception of periostitis, visceral lesions are rare.

### Northern Sudan.

As we travel north down the Nile the country, climate and people change. At Malakal, in the Upper

PLATE XVIII

Fig. 1.
The secondary lesion of yaws.

Fig. 2.
A secondary yaws lesion which has healed centrally and become annular.

Fig. 3.
Gangosa.

Fig. 4.
Acquired syphilis in an infant. Note lesions on the mother's breast due to inoculation from the suckling infant.

PLATE XIX

Fig. 5.
Axillary chancre and fine papular syphilide.

Fig. 6.

Profuse crusted secondary syphilide in a desert Ar[illegible]

Fig. 7.
Perineal condylomata of the late secondary stage.

Fig. 8.

Secondary annular syphilide on neck of Khartoum prostitute.

Nile province, the tribes are different and a larger proportion of the people wear clothing. Here the disease is rather more frequently acquired in adult life, although not necessarily venereally: extra-genital primary lesions are often seen. Figure 4, taken at Malakal, illustrates a condition seen throughout the Sudan; the child was born healthy and caught the disease from a playmate; the mother had some lesions on her breast, acquired from the child, but she had not developed any generalized lesions. It seems likely that this partial immunity of the mother may be due to previous infection in her own childhood.

In the desert of the north-west Sudan, in Kordofan and Darfur provinces, the population consists of a mixture of tribes, many of whom are nomad Arabs. The climate is hot and dry. The natives wear clothing to protect them from the sun. In this part of the country *treponematosis* is more akin to syphilis as we know it in Europe. There is a fairly high incidence of venereal infection, with typical Hunterian chancres, in adults, but extra-genital and childhood infection is still very common. Figure 5 shows a chancre in the axilla of a man who has a fine generalized papular syphilide. The cutaneous eruptions are as a rule very florid (Figure 6) and tend to persist in moist areas as condylomata (Figure 7); such condylomata are indistinguishable from those seen in the Bahr-el-Ghazal province in cases of *yaws*, and equally from those of undoubted syphilitics in Khartoum. Periostitis is the most painful feature and is apt to be present for years, giving rise eventually to marked bony deformities. Aneurysms are occasionally seen, but cardio-vascular lesions are far less common than the prevalence of the disease would lead one to expect. Routine examination of the cerebrospinal fluid shows a higher incidence of pathological changes than was found in the south, but considerably less than in Khartoum. Meningo-vascular syphilis is not uncommon, but tabes and general paresis are unknown.

In a country where normal births are not registered it is obviously impossible to form an estimate of the frequency of still-births. Occasionally one sees new-born infants covered with a profuse cutaneous syphilide, but these seldom survive. It would almost seem as though the parents fail to transmit the disease unless they themselves are suffering from a recent infection: in this case the fœtal infection is so heavy that it is lethal. I have heard of only one case of late congenital syphilis in the whole Sudan; this was seen by one of my medical colleagues in Darfur: the patient was a man with a sunken nasal bridge and Hutchinson's teeth. Frequently natives say that their tertiary lesions are inherited, but that is only in regions where civilization has made them conscious that some moral stigma attaches to the admission of venereal infection.

### KHARTOUM AND OMDURMAN.

In Khartoum and Omdurman, the largest towns in the Sudan, the population is very mixed. There are Arabs and various Nilotic tribes. The conditions of life are very different from those of the regions we have so far considered. People live in houses and wear complete clothing. The climate is hot but not very humid, and, except during the brief rainy season, the ground is parched by the sun. Syphilis is very prevalent: thirty per cent. of the 400 prisoners in the Central Prison in Khartoum, whose blood I examined, had positive Kahn reactions. It is almost entirely venereally acquired and typical chancres on the genitals are the rule. Occasional chancres on the lip and tongue are not really exceptions to the venereal rule. The secondary stage is characterized by various types of cutaneous syphilide, most of which are much more florid than anything seen in England to-day. Figure 8 shows the neck of a Khartoum prostitute in the secondary stage ; the papules are healing in the centre and thus becoming annular. They are precisely similar to those seen in the same stage of *yaws* in the southern Sudan. Crusted *frambœsial* lesions are also seen, but not so commonly as in the south. The secondary skin lesions clear up spontaneously, more rapidly than they do in the south, and there is, therefore, more distinction between the secondary and tertiary stages. Lesions on the mucous membrane of the mouth are more commonly seen. Periostitis is still responsible for most of the patients' discomfort.

In the tertiary stage aortitis is common. I was able to collect a good series of specimens of aneurysm of the aorta for the pathology museum of the Kitchener School of Medicine. Gummata are of fairly frequent occurrence. I made a neurological study of over four hundred cases of syphilis in the district of Khartoum and also examined every lunatic who was held in custody. My results showed that there is a heavy invasion of the meninges, as judged by gross changes in the cerebrospinal fluid, in the secondary stage. Sometimes this is sufficient to give symptoms of meningitis with ocular disturbances. Such cases of clinical meningitis occur especially as neuro-recurrences some six months after the cessation of insufficient arsenical treatment. In the first six months forty per cent. of cases had pathological cerebrospinal fluids ; this figure then fell until the fifth year, when it again rose, reaching about thirty-five per cent. in fifteen years.

These figures do not mean that thirty-five per cent. of tertiary syphilitics have neuro-syphilis, but that spirochætes have reached the meningo-vascular supporting structures of the central nervous system. Thirteen per cent. of 229 tertiary syphilitics admitted to hospital were actually being treated for meningo-vascular syphilitic symptoms, and yet there has never been a single case of tabes or general paresis in any native of the Sudan! I examined every lunatic who was in custody, and there was never a case of general paresis. On the other hand both tabes and general paresis occur in Greeks, Egyptians and other nationalities living, and presumably infected, in the country.

There is clear evidence in Khartoum that congenital transmission does occur, and it is probably true to say that a large number of still-births are due to that cause; and yet even here one does not see the classical stigmata of congenital syphilis.

### MOROCCO, ALGIERS AND EGYPT.

In Northern Africa, Morocco and Algiers, syphilis manifests itself in the same way as it does in the desert regions of the north-west Sudan. Lacapère has described the profuse crusted cutaneous eruptions of the secondary stage, the paucity of tertiary visceral lesions and the absence of tabes and general paresis. Here again childhood infection is common.

In Egypt syphilis is much more as we know it in England: it is almost entirely venereally acquired; the secondary syphilides are less florid than they are in the Sudan, but still occur more constantly than they do in this country. Visceral tertiary manifestations are frequent and tabes and general paresis are found: the last less commonly than in England.

### SYRIA.

In Syria great differences have been noted by Hudson[3] between the manifestations of syphilis as it occurs in town-dwellers and in the nomads of the surrounding desert. So great is the difference that the Arabs consider the desert form to be a distinct disease and give it a separate name *bejel.* For details of this condition reference must be made to Hudson's description, and it suffices to say that in the towns the disease is venereal syphilis, while in the desert it is a childhood disease. Nomad Arabs frequently come into Omdurman, in the Sudan, and bring their whole families to hospital for anti-syphilitic treatment: the children, of all ages, have as a rule profuse crusted sores; it is one of the exanthemata of childhood and yet it is surely syphilis.

Macqueen[4] has described an identical state of affairs in Palestine, and recently von Düring[5] has published his observations on endemic syphilis in Constantinople and the north-western provinces of Asia Minor between the years 1890 and 1902: here again it was a childhood disease with few visceral lesions in the tertiary stage but many disfiguring involvements of the palate and nasal bones, akin to gangosa.

In the hinterland of Dalmatia, Bosnia and Herzegovina the same type of childhood infection has been reported, and it is interesting to note that up to a hundred or so years ago there was an endemic disease in the north of Scotland called *sibbens* which seems to have been non-venereal syphilis. Ehlers says that the same thing was true of Denmark, and that in Sweden one hundred years ago ninety per cent. of cases of syphilis were acquired non-venereally.

In many other tropical countries, such as Jamaica and the Philippines, yaws occurs. There has recently been a Royal Commission on Yaws in Jamaica and in their report Turner[6] and his associates state their belief that yaws and syphilis are distinguishable: but they do so on the basis of the same criteria that appear in Manson-Bahr's table, and this to my mind invalidates their conclusion.

It might be thought that one should be able to make a distinction between yaws and syphilis by histological examination of biopsy specimens of the skin lesions. I have excised many lesions of all types from different parts of the Sudan and am quite unable to detect any differences between comparable lesions of yaws and syphilis. The much-quoted *endarteritis* is by no means a salient feature of the secondary skin lesions in syphilis, and there is certainly no justification for making that one of the criteria for differential diagnosis.

### DISCUSSION.

We have now made our brief survey of *treponematosis* in many parts of the world. It remains to attempt an explanation of the differences we have observed.

It is surely clear that people who wear no clothing and have no knowledge of personal hygiene have ample opportunities for acquiring syphilis without sexual intercourse: it follows that the site of the primary lesion and the manner in which it is acquired are of no significance. But, nevertheless, there is one important aspect of this question: venereal disease is acquired by adults. When an infection is transmitted by ordinary contact children are at risk. I am sure that this is the explanation of the frequency of yaws in childhood; and, since the disease is very prevalent in the districts where it occurs, a high proportion of the population is infected in childhood and thus become immune to subsequent adult exposure. Blacklock[2] has stressed the importance of distinguishing between syphilis acquired in childhood and that acquired in adult life. Many diseases are different under these two conditions. Some of the results of childhood infection with syphilis can be predicted, and the chief of these is the bearing that it will have upon the frequency of congenital transmission subsequently. It is known that the risk of an untreated syphilitic woman transmitting the disease to her children diminishes very considerably with the passage of time. It is probably safe to say that this risk is slight at the end of ten years: if that be conceded we have an explanation of the rarity of congenital *yaws*, for by the time affected children reach child-bearing age they are no longer infective. Unfortunately, this is not the whole story, for we have seen that adults as well as children do acquire the disease and they should be capable of transmitting it to their children. We have noted that it is not only in yaws districts that congenital disease is rare: precisely the same condition obtains in syphilis in the northern Sudan and in *bejel* in Syria. I should like to suggest, tentatively, that when syphilis is transmitted through the placenta in these people it produces so great a reaction that the fœtus is killed. We have seen that the disease is characterized by very profuse cutaneous lesions in children and in adults: it is perhaps reasonable to suppose that the fœtus shares this capacity for responding violently to the infection; it is certain that the cases of congenital disease that I have seen have been covered with moist syphilides. It seems to be a case of "all or none."

It would be very useful if we could explain why the cutaneous reaction is so severe in natives of the tropics. A partial explanation, at any rate, is provided by a consideration of the factor of exposure. The most exuberant lesions occur among naked people living in a hot, humid atmosphere. The desert Arabs are exposed to the sun and their scanty clothing is indescribably dirty: they never wash; their cutaneous lesions are second only in severity to those of the last group. And it is interesting to note that when an Arab takes up residence in a town, wears clothes, and learns to wash, his secondary syphilide is much less florid. This, essentially, is the difference, to my mind, between the cutaneous lesions of Hudson's cases of *bejel* and those of his town-dwellers. But here again, that is not the whole story. Europeans living in the tropics show the usual European manifestations when they contract syphilis: there is a racial factor in addition to the environmental one.

We noted at the beginning of this paper that syphilis has suffered a change in Europe since it was first introduced in the fifteenth century. Tropical syphilis to-day has much in common with the great pox. It is tempting to suggest that syphilis was not introduced to the tropical countries where yaws now abounds until a comparatively recent date, but here we are on very unsure ground. Although it is fairly generally accepted that syphilis was brought from America by Columbus the evidence is not by any means complete; proof of pre-Columbian syphilis is not easy to adduce on either side of the Atlantic. The origin of yaws is still more obscure: it was present in Central Africa when the first European explorers arrived there, but that is little more than a hundred years ago, and of its previous history we know nothing.

The suggestion has been made that the absence of tabes and general paresis, and also the character of the cutaneous reactions, may be due to the fact that malaria is endemic. The view that malarial attacks in the early stages of syphilis may have a prophylactic action against the development of general paresis does not seem to be warranted by the evidence. Many cases have been recorded where Europeans living in the tropics have been exposed to almost constant malarial infection before, during, and after their infection with syphilis, and yet in due course they have become paretic. It seems that malaria serves only as a therapeutic measure. It might be argued that the natives do not leave the malarial country in which they live and so are getting the benefit of constant malarial treatment. There is one great objection to this suggestion: adults in highly malarial districts have a considerable immunity to malaria and it is almost only the children who suffer; adults may be infected when they are bitten by mosquitoes, but the plasmodium does not proliferate sufficiently to cause a febrile reaction. One might expect to get valuable information on this point from statistics of syphilis in the American negro, but they are difficult to interpret. There are conflicting reports in the literature with regard to the relative incidence of general paresis in whites and blacks admitted to asylums in the United States. The possibilities of error are self-evident. The proportion of whites to blacks in the contributory population and the relative incidence of syphilis and of other types of mental disease must be taken into account. There are several marked differences between syphilis in the American negro and that in whites, but these negroes cannot be compared with their African cousins because their environment is so different.

The possibility that there are several different strains of *treponema* responsible for the varying grades of syphilis must be considered. Cultural methods are not available and one has to rely upon the results of animal inoculation. Pearce and Brown[7], and Chesney[5] have reported certain differences between the testicular lesions produced in rabbits with *yaws* and syphilis strains, but it is not certain that these are constant. In a small series that I inoculated I could see no difference. Schöbl[9] has described some most striking lesions in Philippine monkeys inoculated with *yaws*: they resembled human *yaws* most surprisingly, but he did not use sufficient controls with other strains of treponema from cases of syphilis. It is sometimes said that there are *neurotropic* strains of the treponema of syphilis which are responsible for general paresis and tabes, but the evidence for this is most unsatisfactory. The original *neurotropic* strain was isolated by Nichols[10] from the cerebrospinal fluid of a case of meningo-vascular syphilis, not from a paretic, so that it has no claim to be considered neurotropic at all. There is no denying that different strains of *Treponema pallidum* do exist: they have quite different effects on experimental animals; but there is very little evidence that they are responsible for the variety of the manifestations in man.

The part played by treatment is a difficult one to assess, and it cannot be discussed here. I believe that treatment in the early stages robs the patient, as it does experimental animals, of a certain amount of immunity, but since that immunity is very slight it is a good bargain to exchange it for thorough treatment. Unfortunately, natives in the tropics seldom get thorough treatment and there is reason to fear that they may be harmed by it. With reference to the statement that yaws differs from syphilis in not responding to mercurial treatment, it is amusing to note that one of Castellani's two cases, from whom he obtained his *Treponema pertenue*, was undergoing mercurial treatment with improvement.

### SUMMARY.

In conclusion, I believe that the chief differences between the reaction of the native of the tropics to the treponema and that of the white man depend upon the fact that the former gets a violent cutaneous reaction in the secondary stage and so acquires a greater immunity. The law of inverse proportions enunciated by Brown and Pearce holds for man as well as for animals, in that the visceral lesions of the tertiary stage are inversely proportional to the severity of the original cutaneous reaction. There is room for further experimental work in animals to determine just how much difference there may be between the various strains of treponema. Most of the work in the past has been done with rabbits. It would be most interesting if someone were to repeat Schöbl's[9] experiments on the Philippine monkey, using strains of treponema from various parts of the world. In the present state of our knowledge it appears that the varying responses to infection that we have just surveyed can best be explained on the basis of racial and environmental factors, and of these the racial is by far the greater.

### REFERENCES.

[1] Manson-Bahr, P., *Manson's Tropical Diseases*, 8th Ed., 1925, 476.

[2] Blacklock, D. B., *Ann. Trop. Med. and Parasit.*, 1932, vol. xxvi, p. 423.

[3] Hudson, E. H., *Trans. Roy. Soc. Trop. Med. and Hyg.*, 1937, vol. xxxi, p. 9.

[4] Macqueen, J., *Brit. Jour. Ven. Dis.*, 1934, vol. x, p. 33.

[5] von Düring, E., *Forschungen und Fortschritte*, 1935, vol. xi, p. 187.

[6] Turner, T. B., Saunders, G. M., and Johnstone, H. M., " Report of the Jamaica Yaws Commission," 1934.

[7] Pearce, L., and Brown, W. H., *Jour. Exper. Med.*, 1925, xli. 673.

[8] Turner, T. B., and Chesney, A. M., *Bull. Johns Hopkins Hosp.*, 1934, liv. 174.

[9] Schöbl, O., *Philipp. Jour. Sci.*, 1928, xxxv. 209.

[10] Nichols, H. J., *Jour. Exper. Med.*, 1914, xix. 362.

## Figures and Tables

**Fig. 1. f1:**
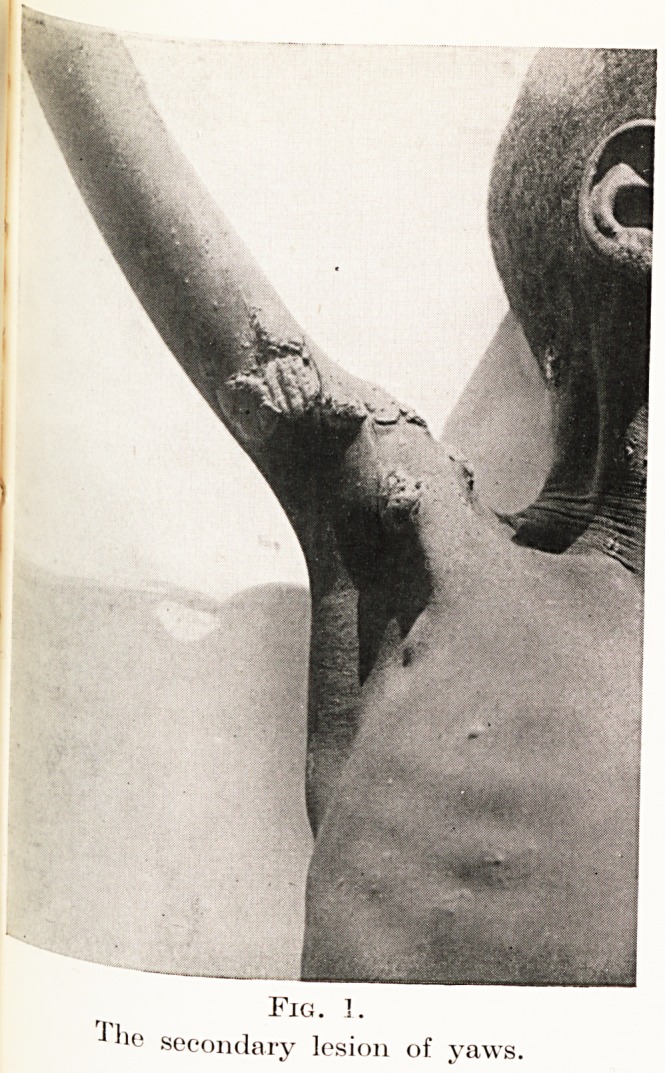


**Fig. 2. f2:**
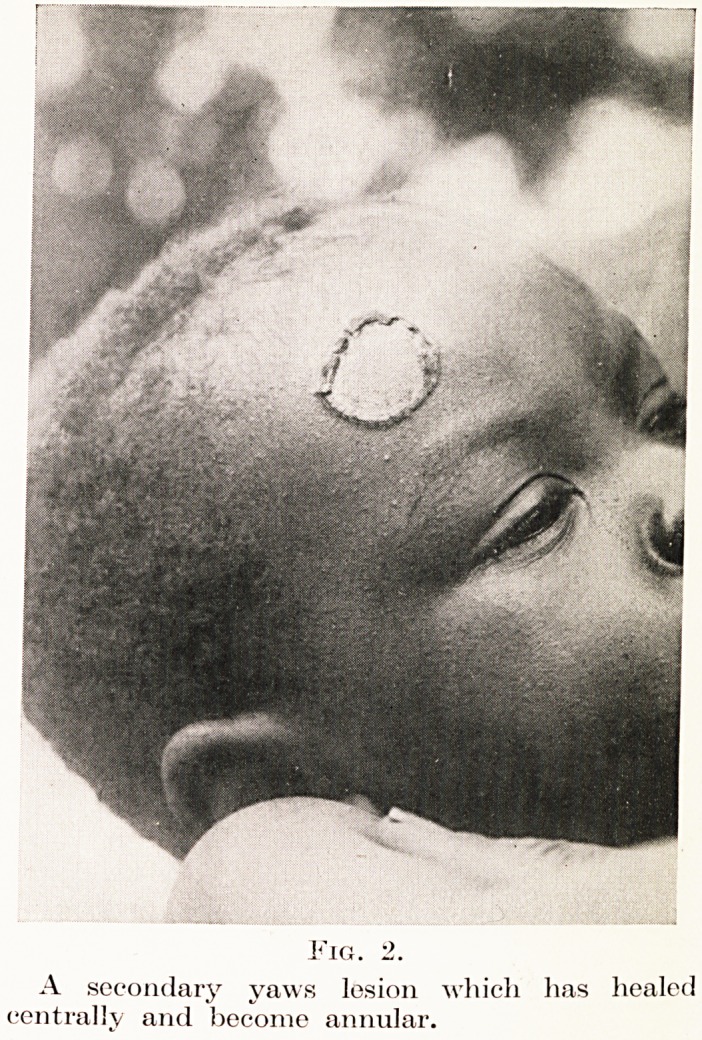


**Fig. 3. f3:**
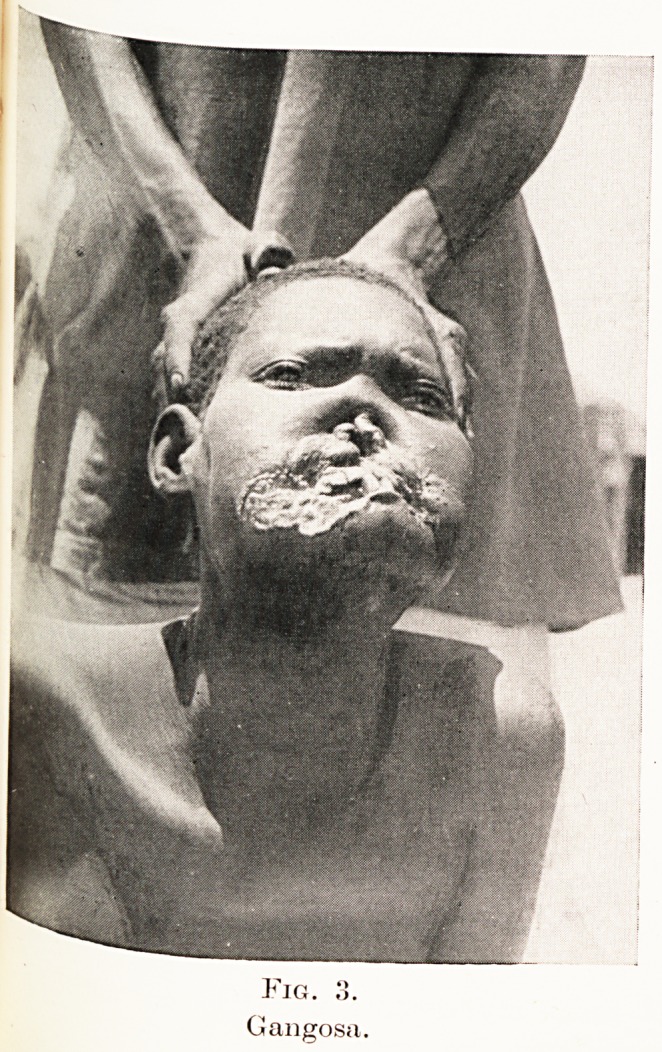


**Fig. 4. f4:**
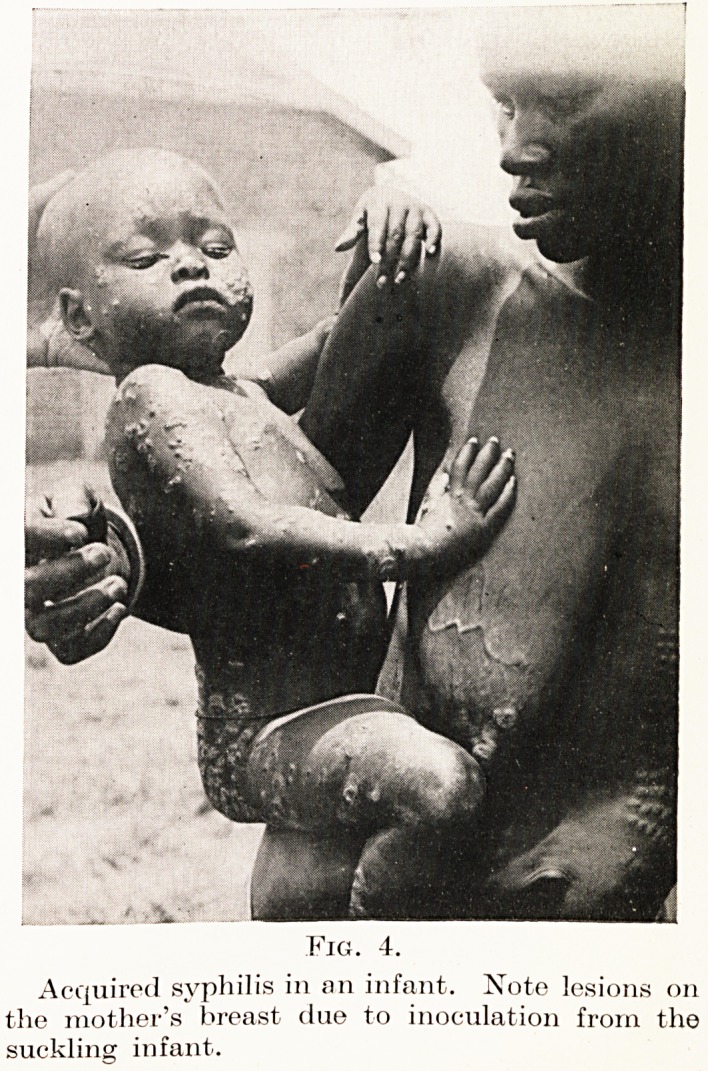


**Fig. 5. f5:**
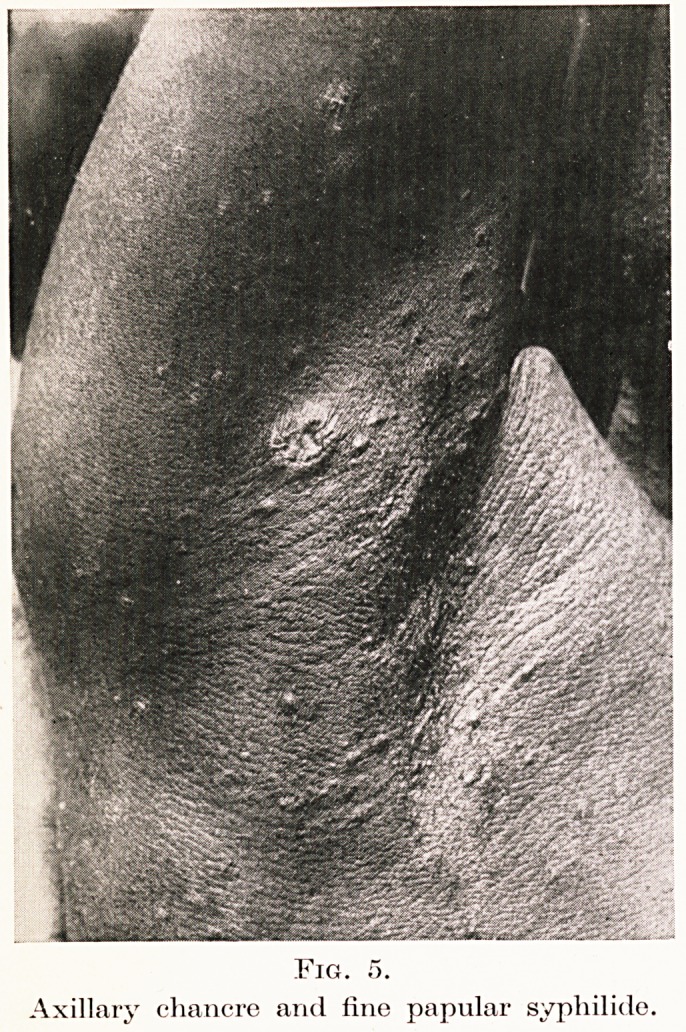


**Fig. 6. f6:**
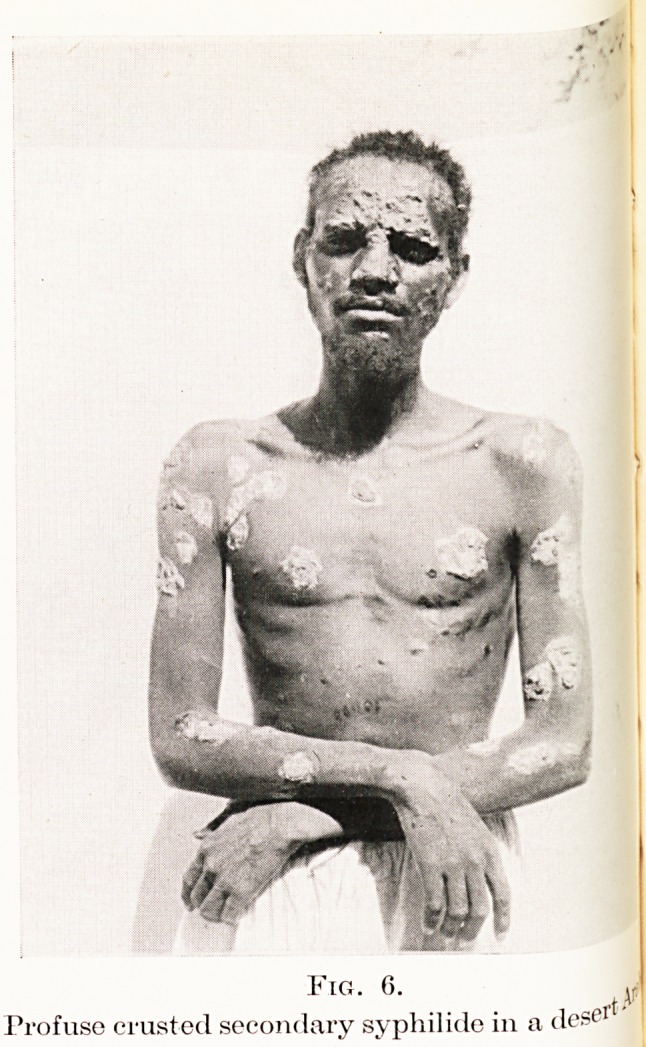


**Fig. 7. f7:**
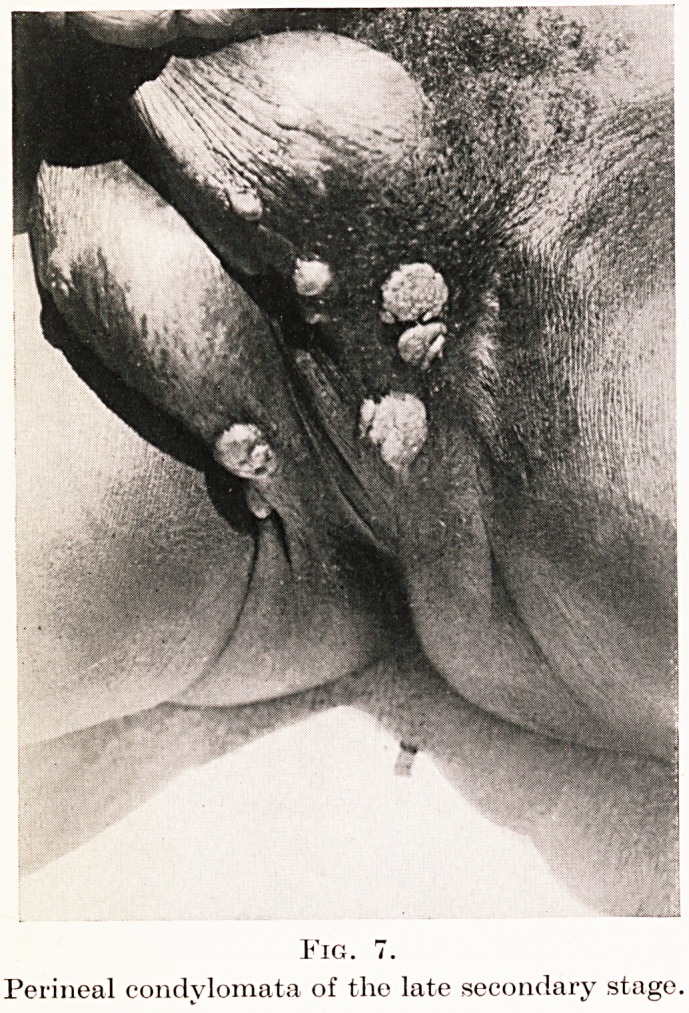


**Fig. 8. f8:**